# The Case for Modernizing Biomedical Research in Ireland through the Creation of an Irish 3Rs Centre

**DOI:** 10.3390/ani12091078

**Published:** 2022-04-21

**Authors:** Viola Galligioni, Dania Movia, Daniel Ruiz-Pérez, José Manuel Sánchez-Morgado, Adriele Prina-Mello

**Affiliations:** 1 Comparative Medicine Unit, Trinity College Dublin, College Green, D02 PN40 Dublin, Ireland; galligiv@tcd.ie (V.G.); ruizpred@tcd.ie (D.R.-P.); 2 Laboratory for Biological Characterisation of Advanced Materials (LBCAM), Trinity Translational Medicine Institute/Department of Clinical Medicine, School of Medicine, Trinity College Dublin, James’s Street, D08 W9RT Dublin, Ireland; dmovia@tcd.ie

**Keywords:** 3Rs principle, new approach methodologies, animal replacement, 3Rs Centre, Irish biomedical research

## Abstract

**Simple Summary:**

The 3Rs principle indicates the measures to refine, reduce, and replace the use of animals in research. Thirteen out of the twenty-seven European countries already have 3Rs Centres. These support the development, acceptance, and/or implementation of methods that address the 3Rs principle at a research and educational level. To date, Ireland has no 3Rs centre. In this commentary, we present the reasons for Ireland to embrace the creation of a national Irish 3Rs Centre. We believe the centre will enable Ireland to be ready for the paradigm shift that is internationally occurring in biomedical research, towards the modernisation and replacement of tests carried out in animals when it is scientifically possible to do so.

**Abstract:**

Since its publication, the 3Rs principle has provided a cornerstone for more ethical and humane biomedical and regulatory research. In Europe, the 3Rs principle has been incorporated into the European Directive 63/2010/EU, with the ultimate aim of fully replacing the procedures on live animals for scientific and educational purposes as soon as it is scientifically possible to do so. Thus, a critical shift in the discussion on animal use in biomedical and regulatory research is undergoing in Europe, a discussion where satisfying the “replacement” principle is becoming more and more defined as a scientific rather than ethical need. 3Rs Centres have been established in recent years across Europe. To date, Ireland has no 3Rs Centre, and the uptake of the 3Rs principle, and in particular of the “replacement” aspect, has been slow. In this Commentary, we present the Irish context of the use of animal models in biomedical and regulatory research, and urge for what, in the authors’ opinion, are the most critical actions that Ireland must undertake to align its biomedical (basic, applied and translational) research with the European 3Rs strategy.

## 1. Introduction

The 3Rs principle, i.e., the reduction, refinement, and replacement of animal use in biomedical (basic, applied, and translational) and regulatory research, has now been published for more than 60 years [1], providing a cornerstone for more ethical and humane biomedical research, as well as today’s careful regulation of the applications of animal testing. As stated by Russell and Burch, the term “reduction” indicates a reduction in the number of animals used to obtain information of a given amount and precision [1]. The reduction in the numbers of animals used must be appropriately designed and analysed, so that animal experiments are robust and reproducible, and truly address a knowledge gap. “Refinement” means any decrease in the incidence or severity of inhumane procedures applied to those animals that still have to be used [1]. Thus, refinement is based on the understanding of the impact of animal welfare on scientific outcomes [2,3], and it includes any method that minimises animal suffering and the incidence or severity of inhumane procedures applied to those animals that still must be used for research. Finally, “replacement” indicates the substitution of higher-conscious living animals with insentient materials [1]. The principle employs methods, models, and tools that allow for addressing scientific questions while avoiding the use of animals. As DeGrazia and Beauchamp put it, the 3Rs principle promotes harm reduction and may be best interpreted today as rules for protecting animal welfare as well as for good science [4].

In Europe, the 3Rs principle has been incorporated into the European Directive 63/2010/EU regarding the protection of animals used for scientific purposes [5]. As stated in paragraph 10 of its introduction, the Directive’s ultimate aim is the full replacement of procedures on live animals for scientific and educational purposes as soon as it is scientifically possible to do so. While the Directive recognises that animals are still needed, procedures should aim to implement the “replacement” principle as much as possible. As stated in Article 1, Directive 63/2010/EU also applies to regulation EC no. 1223/2009 [6,7] relating to the ban of animal testing for both cosmetic products and their raw materials in Europe. Nevertheless, European regulations overseeing animal research are very complex, and present many nuances. To give an example from the regulatory research space, while it is true that in Europe it is illegal to test cosmetic ingredients in animals, substances used in cosmetics may also be used in other consumer products. As such, they are subject to the REACH (Registration, Evaluation, Authorisation, and Restriction of Chemicals) Regulation. Essentially, when a chemical substance is used for other purposes in addition to making cosmetics, animal testing may have to be carried out under REACH to obtain information about the risks involved for those other uses. In addition, REACH may request animal testing to protect the health and safety of workers handling the substances used to manufacture cosmetics. A recent work from the Center for Alternatives to Animal Testing Europe (CAAT-Europe) found that 63 of the 3206 chemical dossiers with cosmetics use reported in vivo tests completed after the Cosmetic Regulation ban on animal testing [8].

On the other hand, in the biomedical space, the European Medicine Agency (EMA), the European Union agency in charge of the evaluation and supervision of medicinal products, is promoting the 3Rs and welfare standards for the treatment of animals in all aspects of the development, manufacture, and testing of human and veterinary medicines [9]. The EMA states that its efforts are in agreement with Directive 63/2010/EU [9]. The use of a standard 3Rs statement for inclusion in all relevant EMA guidelines has recently been agreed upon. The statement reads as follows: “In accordance with the provisions of the European Convention for the Protection of Vertebrate Animals Used for Experimental and Other Scientific Purposes and Directive 2010/63/EU on the protection of animals used for scientific purposes, the 3R principles (replacement, reduction, and refinement) should be applied to the regulatory testing of medicinal products” [10]. EMA’s “Regulatory Science Strategy to 2025” [11] is supported by evidence showing that animal-based preclinical research is associated with critical issues in generating data that can translate to humans [12,13,14,15,16]. This may potentially play a major role in holding back current biomedical (basic, applied, and translational) progress. Accordingly, in February 2018, the EMA published a review of its guidelines, which were updated so as to implement best practice with regard to 3Rs in the regulatory testing of medicinal products [10]. The purpose of this review is to ensure that EMA’s guidelines do not make reference to animal tests that are no longer considered appropriate. Furthermore, on page 20 of its “Regulatory Science Strategy to 2025” document [11], the EMA clearly promotes the reduction/replacement of animal testing and the use of non-clinical models through the “development of clear guidance to encourage and prioritise the use of New Approach Methodologies (NAMs) that can be used to fulfil testing requirements in lieu of traditional animal tests, and that take the 3Rs into serious consideration” [11].

Based on the evidence reported above, it is evident that a critical shift in the discussion on animal use in biomedical (basic, applied, and translational) and regulatory research is occurring in Europe, a discussion where satisfying the “replacement” principle is becoming more of a scientific rather than ethical need for the biomedical scientific community. Even so, discussions on animal replacement are not without controversy. Scientists have often highlighted the benefits that animal research has provided to the advancement of human medical procedures and disease treatment. Clearly, this view has always been strongly rejected by animal-welfare associations, who say that such benefits are irrelevant, even if research on animals could (and has) save many human lives. Even public opinions are divided on animal experimentation [17], because a proportion of the European population does not support animal research, while public demands for treatments for incurable diseases (even when based on animal research for their safety and efficacy testing) are rising. However, based on new evidence becoming available, opposing interpretations of animal use in biomedical research might soon be replaced by a new shared view, where continued animal research is defined as one of the factors currently impeding scientific progress. In line with this new view, a motion has been recently voted on by the European Parliament urging EU countries to accelerate the transition to a research system that does not use animals [18]. Unfortunately, in its response [19] to the abovementioned motion, the European Commission (EC) has failed to provide a list of concrete steps that could eventually lead to the phasing out of animal experiments in Europe. The EC has, however, indicated the intention of strengthening the private−public European Partnership for Alternative Approaches to Animal Testing, so as to build a consensus in targeted areas of regulatory research.

Based on the ongoing paradigm shift in both biomedical and regulatory research highlighted above, 3Rs Centres have been established in recent years across Europe, not only to develop novel predictive non-animal models, but also to disseminate knowledge on alternatives to animal studies, liaise with regulatory bodies and the public, and support the implementation of the 3Rs principle in third-level education, training, and scientific research. Currently, in Europe, there are 11 3Rs Centres; the full list is regularly being updated and is publicly available on the Norecopa website [20]. Moreover, a network of the European 3R Centres (the EU3Rnet) has been established in connection with the EUSAAT conference in 2018. In the consensus statement published in 2020 [21], EU3RNet emphasizes the importance of the developments and dissemination of 3Rs resources at multiple levels: animal caretakers, technicians, veterinarians, teachers, lecturers, and scientists. To date, Ireland has no 3Rs Centre, but the Comparative Medicine Unit at Trinity College Dublin is part of EU3RNet, and it has been actively participating in all of the initiatives and actions from the network with the aim of establishing the first 3Rs Centre in Ireland.

In the following section, we discuss in detail the Irish context of the use of animal models in biomedical (basic, applied, and translational) and regulatory research. With the aim of starting a conversation among the Irish scientific community involved in animal research, as well as in the development/implementation of NAMs, we urge for what, in the authors’ opinion, are the most critical actions that Ireland must undertake to align with the European 3Rs strategy, with particular emphasis on the biomedical research field and the need to establish an Irish 3Rs Centre. In doing so, the authors have not provided insight into how such a Centre should be funded and operated, as we believe this should be discussed at a national level by the Government of Ireland in consultation with the Minister of Health, the Minister of Higher Education, the national funding agencies, and the higher education institutions.

## 2. Discussion

Animal research in Ireland was following the European legislation through the adoption of the European Directive 86/609/EEC. However, it started to be truly regulated mainly with the recent adoption of Directive 63/2010/EU. The latter entered into force in the Irish Law on the 1 January 2013, as SI 543 of 2012 (and its following amendments). Every breeder, supplier, and user of animals for research purposes must fulfil such legislative requirements in Ireland. Nevertheless, the uptake of the 3Rs principle, with particular emphasis on the “replacement” aspect, has been slow in Ireland. Below, we provide an overview of the current status, and we discuss how the creation of an Irish 3Rs Centre could provide a solution to current issues.

The Health Products Regulatory Authority (HPRA) is the Irish competent authority overlooking the application of current national regulations governing the use of animals in research. The HPRA ensures the application of the 3Rs principle at the establishment level, as well as in the ethical authorization process of scientific research projects. According to the HPRA annual statistical report for animals used under the scientific animal protection legislation in Ireland [22], in 2020, almost 75% of animal tests (~103,000 procedures) were carried out for regulatory purposes, i.e., to satisfy the legal requirements for producing, placing, and maintaining products/substances on the market, including safety and risk assessment for food and feed. Approximately 16% of animals (~21,500 procedures) were used for applied or translational biomedical research, followed by almost 8% (~10,500 procedures) for basic biomedical research, and approximately 2% (~3000 procedures) for studies on the protection of the natural environment in the interest of the health or welfare of human beings and animals.

Looking at the data above, regulatory testing appears as the main field where the implementation of new approach methodologies (NAMs) could have a real impact in the uptake of Directive 63/2010/EU and the achievement of its ultimate aim in Ireland. In fact, compared to the European trend, Ireland has an incredibly high number of animals used for regulatory research. According to the data available on the European statistic database on the use of animals for scientific purposes (ALURES), in 2018, only 23% of animals were used for regulatory research in Europe [23]. Such a difference between the European and Irish trends is associated with the presence of a large, preclinical contract research organization (CRO) (e.g., Charles River Laboratories) operating in Ireland for its global market, affecting the national animal return data. The vast majority (>99%) of the regulatory tests for which animals were used in 2020 in Ireland, in fact, were performed on medicinal products manufactured for use in humans [22]. Nevertheless, compared to previous years, 2020 saw a 29% (compared to 2018) and 47% (compared to 2017) decrease in the total number of animals being used in Ireland for regulatory testing [22]. This reduction, as stated by the HPRA in its report, is “due to the ongoing transition from animal tests to non-animal alternatives” [22]. The development of “replacement” strategies/methodologies for regulatory research is in fact more advanced than in the biomedical (basic, applied, and translational) research fields, with several databases of validated NAMs for regulatory testing being publicly available for consultation. These include, for example, the DB-ALM (Database on Alternative Methods) [24] produced by the European Union Reference Laboratory for Alternatives to Animal Testing, and the TSAR (Tracking System for Alternative methods towards Regulatory acceptance) [25].

On the other hand, if we analyse the use of animals in biomedical (basic, applied, and translational) research, and we compare the national efforts to those of other European countries (e.g., Denmark and The Netherlands), Ireland has not undertaken any valuable initiatives for becoming a leader in human-relevant biomedical science. National funding agencies emphasise their commitment to the 3Rs principle, recognising the need to replace animal experiments with cutting-edge, human-relevant technologies in basic, applied, and translational research. Science Foundation Ireland (SFI), for example, has implemented a specific policy related to animals used in biomedical research, where applicants are expected to strictly adhere to the general principles of Directive 63/2010/EU. In addition, it supports the Joint European Funding Principles for Research involving Animals [26]. Despite this commitment, however, no national agency currently financially supports the development and/or validation of NAMs, and current Irish biomedical (basic, applied, or translational) research is still heavily based on animal testing. Thus, we believe that national efforts should focus initially on aligning the biomedical research field in Ireland with the European 3Rs strategy. Ireland is one of the few European member states missing a 3Rs Centre. This raises several issues in enabling the strategic alignment we advocate for, as discussed in detail below.

Firstly, in Ireland, institutional ethics committees are responsible for reviewing the proposed use of animals at a local level, prior to mandatory HPRA authorisation request [27]. Ethics committees are instrumental in ensuring the implementation of the 3Rs principle in biomedical research projects. They consider the study design, procedures planned, and evaluate situations where there might be a risk that the use of animals could conflict with their best welfare interests. The HPRA encourages the pre-approval of projects by local ethics committees [27]. This is seen to be in the best interests of animal welfare and in the overall efficiency of the process. According to HPRA, local ethics committee should be formed by the Animal Welfare Body and the designated persons (Animal Welfare Officer, Designated Veterinarian, Information Officer, and Training Officer) at the establishment level, one or more representatives of the research community with animal-research expertise, a public representative, and a statistician [27]. However, most of the time, members of local ethics committees have multiple roles within their institutions, and they cannot focus solely on ensuring animal welfare and/or the stringent implementation of the 3Rs principle in animal research authorisation requests. Furthermore, experts in NAMs are not among the participants recommended by HPRA for local animal research ethics committees [27], and, therefore, expert advice on “replacement” may not be available while reviewing research authorisation requests at Irish institutions. In addition, as highlighted by DeGrazia and Beauchamp, the 3Rs principle considers only the animal welfare that is associated with the scientific research procedures applied [4]. It “omits important ethical considerations pertaining to human social benefit, including the likelihood of achieving benefit through animal studies and whether and how the prospect of benefit justifies anticipated costs and harms of research” [4]. Expertise in the implementation of the framework of principles described by DeGrazia and Beauchamp in their book [28] are not always available to local animal research ethics committees. The shortfalls mentioned above negatively affect the extent to which the implementation of the 3Rs principle is discussed when authorising animal research in Ireland. The result is that, although more and more NAMs are available and being developed worldwide, in Ireland, the implementation and uptake of NAMs in biomedical (basic, applied, and translational) research has been limited and slow, and the requirement to meet the 3Rs principle is sometimes treated by researchers as a tick-box exercise. To try to address this issue, the HPRA December 2021 Regulatory update shared with Individual Authorization Holders now requires Irish-based researchers to consult the EURL ECVAM databases of non-animal models for immuno-oncology [29], breast cancer [30], and respiratory [31] and neurodegenerative [32] disease research when applying for amendments or new project authorisation requests for biomedical research. The databases must be consulted in an attempt to identify potential non-animal alternatives, and to demonstrate the need for animal experimentation [33]. Thus, it is mandatory to indicate the search criteria used to consult the abovementioned databases. Applications for studies submitted to the HPRA and without evidence of this database search are returned to the applicant without approval. Indeed, having an Irish 3Rs Centre would provide a centralised facility where knowledge of all published databases is available, offering researchers the tools needed to comply with the current and future HPRA requirements. This would also provide a national centre with expertise to train, support, implement, and advance the 3Rs principle in their research, including the “replacement” principle, through the adoption of existing NAMs in research projects submitted for ethical approval, or the development of new ones where applicable/necessary. To support the creation of such a centre of expertise, we believe a database of the academic experts in NAMs and bioethical questions operating in Ireland in both the regulatory and biomedical research fields should be developed as one of the first key actions of the Irish 3Rs Centre. Such a database will support the work of local ethics committees at Irish institutions by providing the contact details of the NAM experts to be appointed as committee members, to provide specific expertise on “replacement”. This will ensure a thorough, expert, robust, and independent assessment is performed, in compliance with the 3Rs principle. The database could be further expanded in the future to include all stakeholders, such as representatives from industry and animal welfare groups, willing to donate some of their time to the local review of animal-based project authorisation requests.

Secondly, implementation of the 3Rs principle in scientific projects suffers from the fact that, currently, in Ireland, there is no platform where scientists with experience in designing robust animal experiments can interact with scientists developing and using NAMs. The lack of interaction between these researchers hinders the ability to demonstrate the value of NAMs in biomedical (basic, applied, and translational) research and negatively affects the modernisation of biomedical research in Ireland. The Irish 3Rs Centre could enable the knowledge exchange needed between these researchers. In addition, involving researchers carrying out animal research will ensure that the Irish 3Rs Centre covers the wider aspect of the 3Rs principle. It could be argued that in the 21st century, emphasis should be placed only on better science without animals (the full “replacement” or “1R principle”). Nevertheless, we believe that for the success of a national Irish 3Rs Centre, it is deeply important to establish an inclusive environment by considering the need for “refinement”, “reduction”, and “replacement”.

Thirdly, Irish-based researchers developing or implementing NAMs work in isolation in their respective fields (e.g., oncology, psychology, artificial intelligence, and toxicology), and quite rarely do these scientists cross-interact. This is linked to the fact that Irish-based researchers developing NAMs do not have access to a platform where to network with other NAM-focused scientists based in the national territory, leaving them unable to establish national collaborative studies of a high translational impact in the “replacement” field. The presence of an Irish 3Rs Centre would indeed facilitate the highly needed knowledge exchange, interaction, communication, and dissemination of information that can make a difference in putting the 3Rs principle into practice for directly impacting and progressing biomedical sciences. For example, by using recent initiative of the UK National Centre for the 3Rs (NC3Rs) as template [34], the Irish 3Rs Centre could provide resources bringing together activities on NAMs carried out in the national territory. As well as showcasing a portfolio of projects in this topic area, these resources could provide information and support for both the national and international biomedical community, including technology developers, end-users, regulators, and in vivo/in vitro researchers who are looking to adopt or develop NAMs. Furthermore, the Irish 3Rs Centre could play a direct role in leading projects to increase confidence in the use and application of NAMs for biomedical research.

Fourthly, the use of systematic reviews and meta-research methods, which are valuable tools in scrutinizing the validity of biomedical (basic, applied, and translational) research and in properly designing preclinical experiments [35,36], is not broadly spread in Ireland. Menon et al. [37] recently described the relevance and benefits of conducting preclinical systematic reviews, demonstrating that these can positively contribute to the quality of preclinical research in the long-term. The team also identified the need for training and coaching the scientific community on systematic reviews/meta-research methods. The Comparative Medicine group at Trinity College Dublin, of which some of the authors are part, has organised training for scientists on systematic reviews of animal experiments, and it is ambassador of the Systematic Review Centre for Laboratory Animal Experimentation (SYRCLE). Nevertheless, an Irish 3Rs Centre could indeed have a real impact in this area by providing centralised, ad hoc training for all Ireland-based researchers to routinely implement this powerful tool in their preclinical work. Similarly, an Irish 3Rs Centre would have a pivotal role in the establishment of education, training, and CPD programmes for scientists and professionals working in the biomedical fields in Ireland. These programmes could incorporate the numerous existing modules on the implementation of the 3Rs principle, such as those offered by the Education and Training Platform for Laboratory Animal Science (ETPLAS), an EU-wide information portal on education and training.

## 3. Conclusions

Ireland needs to get ready for the paradigm shift that is occurring in biomedical research in Europe, or its researchers will be left behind. As demonstrated by its December 2021 Regulatory update, HPRA will slowly transition to “replacement”, making sure that animal-based procedures are only performed where there is no NAM available, and where the expected benefits outweigh any potential harm to the animals. Local ethics committees must therefore ensure the 3Rs principle is applied to all authorised projects thorough an expert and robust assessment. In doing so, the support of an Irish 3Rs Centre could be pivotal by providing access to knowledge sources on existing NAMs by developing a database on the “reduction”/“replacement” expertise available in Ireland, helping scientists to find information on the 3Rs relevant to their work, and offering advice, networking, and training opportunities for researchers.

With world-class universities and a strong industrial R&D presence on the national territories, Ireland is in a strong position to lead the way in developing NAMs and high-tech alternatives to animal tests. As well as benefitting patients and protecting animals, modernising the Irish biomedical research has a huge economic potential for Ireland. For example, the global 3D cell culture market was estimated to be worth $892 million in 2019, rising to $1846 million by 2024 [38]. In addition, healthcare systems are at a crossroads following the COVID-19 pandemic, which forced a reappraisal of what is truly necessary to deliver safe and effective health solutions as quickly as possible. The speed at which SARS-CoV-2 vaccines were developed was partly due to decision-makers agreeing that human trials could be conducted in parallel with (and sometimes ahead of) animal tests. NAMs can support the transition in policy and practice needed to make today’s healthcare sustainable. For example, NAMs could facilitate national pharmaceutical innovation and competitiveness. Based on a recent market report [39], NAMs may generate an average total R&D cost reduction of 10–26% in the development of new drugs, resulting in an absolute cost saving of up to $700 million per compound, if assuming an R&D cost equal to $2.7 billion per drug. NAMs can therefore impact the ability of the national Irish healthcare system to subsidize the newest, most effective treatments. Ireland has a complex two-tiered national healthcare system, where primary care is only partially publicly funded. The National Centre for Pharmacoeconomics (NCPE) is the government agency responsible for deciding which drugs are subsidized publicly. In its decision-making, the NCPE considers the therapeutic benefit of the agent, but also its cost-effectiveness. Decreasing drug costs will positively affect the number of new reimbursed treatments available to Irish patients. This will reduce the financial and societal impact of diseases on patients and their families.

However, if the implementation of the 3Rs principle is to succeed in Ireland, enabling the positive impact described above on the Irish economy and society, then it is important that the Irish 3Rs Centre is structured in a way that will accelerate such implementation. Its organisation must be well-informed about European initiatives, willing to learn from the lessons learnt by other 3Rs centers, and ready to seek out opportunities to collaborate with them. For example, what stands out from Denmark’s approach to the implementation of the 3Rs principle is its strong emphasis on giving animal protection organisations a meaningful voice within the regulation of animal research. In fact, four members of the Danish Animal Experimentation Council were appointed after consulting the Animal Welfare Organizations [40]. We believe that, although their involvement is often ostracized by animal researchers, animal-protection organisations can provide invaluable input on techniques that can be used to replace animals. By involving such organisations, the Irish 3Rs Centre will accelerate the highly needed modernisation of biomedical research in Ireland. Furthermore, bold policy actions are required in Ireland to establish the implementation of the 3Rs principle and to address the need to transition to “replacement” as a matter of national importance. Policy actions must be informed by strategy roadmaps that the Irish 3Rs Centre could have a key role in developing, with a view to ensure that any gaps in animal-replacement methods are addressed. In drafting the roadmaps and through collaborations with the HPRA, the Irish 3Rs Centre should indeed make sure that Ireland applies for the Animal Protection Index. The index assesses countries against a range of animal protection criteria, giving a ranking of A–G for overall animal protection and ratings for specific areas. Obtaining an Animal Protection Index will provide a quantifiable key performance indicator (KPI) of national legislation and policy commitments to protecting animals, and to measure the success of the roadmaps drafted by the Irish 3Rs Centre. Such roadmaps could also inform strategic funding decisions from national funding agencies, universities’ research strategies, and government investments. The latter could also provide funding for the long-term sustainability of the Irish 3Rs Centre, following the example of the UK Department for Business, Energy, and Industrial Strategy (BEIS), which funds the NC3Rs.

In the authors’ opinion, the Irish 3Rs Centre will have to focus on several key aspects from the start, including the following:

- The development of new and the provision of existing educational programmes and educational resources for undergraduate, postgraduate, and continuous professional education. Novel educational material could include the provision of practical support and training for scientists to reduce or replace the use of animals in their research, and/or to pursue an animal-free career pathway.
- Identifying the national priorities to reduce animal use in biomedical (basic, applied, and translational) research and subsequently promoting the implementation of NAMs in such research fields during the project authorisation process. This will be achieved by providing a platform for knowledge exchange between researchers using animal and non-animal methodologies, as well as by providing access to existing databases and training on in vitro complex culturing techniques, artificial intelligence, and adverse outcome pathways (AOPs).
- Implementing the use of systematic reviews and meta-research methods in preclinical research across Ireland through training and technical support.
- Providing guidance to scientists for improving the readiness and quality of the in vitro and in silico NAMs they develop towards regulatory validation and intellectual property protection.

Following the example of The Netherlands, the Irish 3Rs Centre will need to bring together all stakeholders to facilitate this process, including representatives from government, industry, academia, animal researchers, and animal welfare organisations. Interestingly, depending on how Directive 2010/63/EU has been implemented in the different countries of interest, European 3Rs Centres show very diverse organisational structures, each with their own setup and milestones linked to 3Rs adoption in their respective national agendas. Although at a slower pace than originally planned [41,42], it is the authors’ opinion that The Netherlands have undertaken some of the most valuable initiatives and collaborative approaches in Europe in the 3Rs space, thus providing a valuable inspiration for the establishment of the Irish 3Rs Centre. By fulfilling its functions, the Irish 3Rs Centre will become a key enabling platform that stimulates innovation and growth in Ireland, and more in general in Europe, in a variety of biomedical fields.
